# A Sentinel-2 dataset for railway presence classification across metropolitan France

**DOI:** 10.1016/j.dib.2026.113104

**Published:** 2026-07-23

**Authors:** Lukas Pasold, Luca Eisentraut, Ricardo Buettner

**Affiliations:** Chair of Hybrid Intelligence, Helmut-Schmidt-University/University of the Federal Armed Forces Hamburg, Holstenhofweg 85, 22043, Hamburg, Germany

**Keywords:** Remote sensing, Infrastructure, Land cover, Railway, Computer vision, Satellite imagery, Classification, Railtrack

## Abstract

This article presents a Sentinel-2 image dataset for railway presence classification across metropolitan France. The collection contains 10,000 true-color RGB PNG chips with equal representation of railway and railway-absent scenes. Each chip has dimensions of 224 × 224 pixels and covers a ground footprint of 512 m × 512 m.

Railway chip locations were derived from retained OpenStreetMap railway=rail geometries. The railway class was constructed through geographically balanced sampling along the retained network and distance thinning between selected locations. Railway-absent chips were generated in the local surroundings of railway locations. A railway-absent chip was included only when its complete footprint did not intersect retained railway geometries and did not overlap an already accepted chip footprint.

The image collection was generated from Copernicus Sentinel-2 Level-2A observations acquired by the Sentinel-2 satellite constellation. Bands B04, B03 and B02 were used to create the red, green and blue image channels. Retrieval covered the period from 23 June 2025 to 23 June 2026 and applied a maximum cloud-cover threshold of 10 percent together with least-cloud-cover mosaicking.

The included files comprise the image collection and a CSV metadata table containing image filenames, class labels and stable candidate identifiers. The dataset provides material for railway-presence classification, comparison of image-classification architectures, spatial evaluation designs, and geospatial representation-learning workflows.

Specifications Table

**Table:** 

Subject	Computer Sciences
Specific subject area	Railway presence classification in Sentinel-2 true-color imagery over metropolitan France.
Type of data	Image (RGB PNG image chips), Table (CSV metadata table).
Data collection	Railway candidate locations were derived from OpenStreetMap line geometries tagged railway=rail. Sentinel-2 Level-2A imagery was retrieved through the Copernicus Data Space Ecosystem Sentinel Hub Process API. Railway samples were selected from retained railway geometries using spatial balancing and distance thinning. Railway-absent samples were generated in neighboring chip locations subject to geometric exclusion of retained railway segments and non-overlap with railway chips or previously accepted railway-absent footprints.
Data source location	Metropolitan France. Satellite imagery originates from the Copernicus Sentinel-2 Level-2A collection, providing observations from the Sentinel-2 satellite constellation. Railway and contextual land-cover geometries originate from OpenStreetMap.
Data accessibility	Repository name: A Sentinel-2 Dataset for Railway Presence Classification Across Metropolitan France Data identification number: 10.17632/f2m5jwrjss.1 Direct URL to data: https://data.mendeley.com/datasets/f2m5jwrjss/1
Related research article	none

## 1 Value of the Data

• The dataset enables **robust and reproducible benchmarking of geospatial computer vision methods** based on satellite imagery through a **large-scale, balanced collection** of Sentinel-2 RGB image chips with representations of railway and railway-absent scenes. This design supports binary image-classification models **without class-imbalance effects** dominating performance metrics. All chips share a fixed geographic footprint, image size, and acquisition protocol, enabling controlled comparisons across model architectures and training strategies.
• The dataset captures the **environmental diversity** of real-world railway infrastructure through **geographically balanced sampling** across metropolitan France and **distance thinning** to reduce overrepresentation of dense urban networks and major rail corridors. It includes railway scenes across agricultural and open vegetation areas, urban environments, forests, industrial areas, and water or wetland contexts and is therefore suitable for the evaluation of **model robustness and generalization across heterogeneous environmental conditions**.
• The dataset provides realistic and challenging model evaluation by generating **railway-absent samples in the local surroundings** of corresponding railway locations rather than randomly sampling distant regions. A railway-absent chip was accepted only when its complete footprint did not intersect any retained railway geometry and did not overlap accepted sample footprints. These **contextually similar negatives** make the classification task more challenging and reduce the risk that models rely only on broad regional or land-cover differences.
• The dataset provides a **reusable benchmark** for geospatial deep learning research on **critical transport infrastructure** and enables progress in railway-presence mapping, comparisons of conventional computer-vision models and geospatial foundation models, spatial cross-validation, domain-shift experiments, representation learning, and hard-negative mining. As railways are part of critical transport infrastructure, the dataset may also assist research on **scalable infrastructure inventorying**, large-area screening, and the development of data-driven approaches for infrastructure monitoring.

## 2 Background

Satellite imagery and machine learning enable spatial information on built environments and infrastructure to be derived at scales difficult to address through manual interpretation [1]. They support infrastructure inventorying, large-area screening, temporal comparison, and transferable geospatial models [2,3]. Railway corridors are a relevant application because they form extensive linear networks across urban, industrial, agricultural, and natural settings.

Recent publications demonstrate the value of task-specific Sentinel datasets for reusable machine-learning research, including road detection for infrastructure monitoring, multimodal image collections, and cloud and cloud-shadow benchmarks [[4], [5], [6]]. However, no published Sentinel-2 dataset has specifically targeted railway-presence classification through geographically balanced railway sampling and locally generated railway-absent hard negatives.

Detecting railways in Sentinel-2 imagery remains challenging: individual tracks are narrow relative to the native 10 m resolution [7,8], while vegetation, adjacent roads, station areas, ballast, shadows, and surrounding land use affect their appearance. Robust evaluation therefore requires railway-centered scenes and visually related non-railway surroundings.

This dataset provides a transparent, reusable benchmark for railway-presence classification, supporting model comparisons, spatial validation, representation learning, and analyses across environmental settings.

## 3 Data Description

The dataset [9] consists of Sentinel-2 image chips and an accompanying comma-separated values (CSV) metadata table. As summarized in Table 1, the image collection contains 10,000 true-color image chips stored as RGB Portable Network Graphics (PNG) files, together with one master metadata table containing one record per image. Each image has dimensions of 224 × 224 pixels and corresponds to a 512 m × 512 m geographic footprint in metropolitan France. The images were rendered from Sentinel-2 Level-2A bands B04, B03, and B02, corresponding to the red, green, and blue channels, respectively [7]. Since these bands have a native spatial resolution of 10 m [7], the distributed PNG files represent resampled true-color renderings of the defined ground footprint rather than imagery with a native spatial resolution finer than 10 m.

**Table 1:** Components of the dataset.

Component	Format	Content
Image collection	RGB PNG	10,000 Sentinel-2 true-color image chips, each with 224 × 224 pixels and a 512 m × 512 m ground footprint.
Metadata table	CSV	Image filename, class label, and stable candidate identifier.

The images have a resolution of 224×224 pixels due to the native ground resolution of the Sentinel-2 B-band, which has a ground sampling distance of 10 m. The spatial information encoded in the images is determined exclusively by this resolution, so that exporting them at a higher pixel count (e.g., 512×512) would not provide any additional information but would merely result in denser upsampling. Since the problem being addressed is railway presence classification, that is, the classification of the scene as a whole, the spatial context encoded in the 224 × 224-pixel images is more critical than the representation of finer pixels generated by interpolation. Furthermore, the selected resolution corresponds to a widely used input size for modern CNN and Vision Transformer architectures, allowing the image chips to be used directly with commonly applied and pre-trained models without an additional resizing step. This ensures a consistent input representation across architectures while avoiding unnecessary computational overhead and potential interpolation artefacts.

**Fig. 1:**
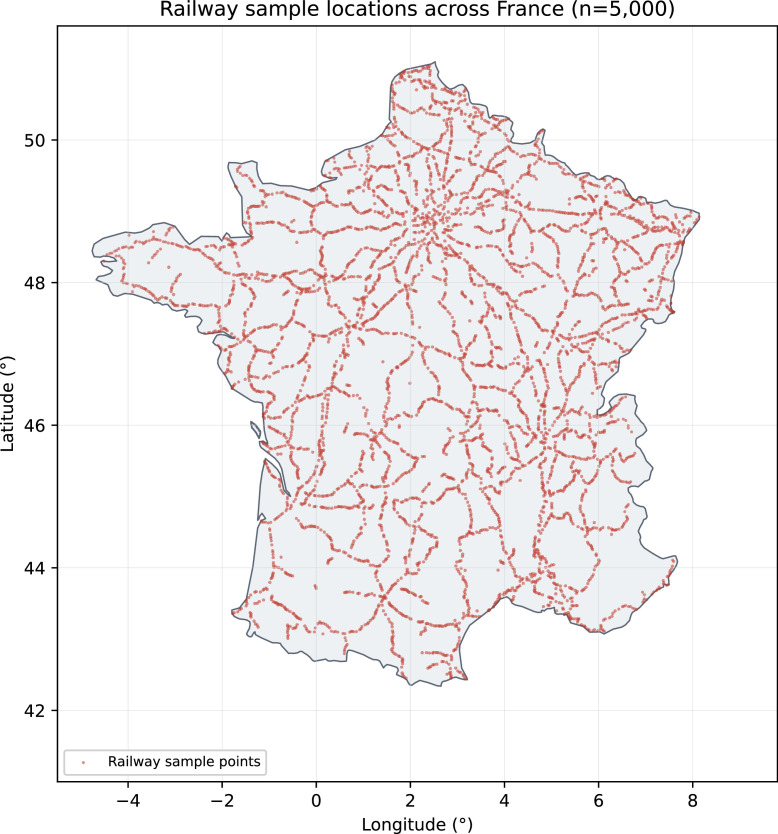
Geographic distribution of the 5,000 railway image-chip centers across metropolitan France. Each point denotes the WGS84 center coordinate of one railway image chip.

The image collection comprises two equally sized classes (5,000 chips labeled railway and 5,000 chips labeled no_railway). Images in the railway class are associated with retained railway geometries, whereas images in the no_railway class were assigned to the railway-absent class according to the geometric criteria described in the Experimental Design, Materials and Methods section. Fig. 1 shows the geographic distribution of the 5,000 railway chip centers across metropolitan France. Railway-absent chip centers are not shown because they were generated in the immediate vicinity of the corresponding railway locations and would substantially overlap with railway locations at the map scale.

The master metadata table links every image filename to its class label, and stable candidate identifier. The structure of the metadata table is illustrated below using an example record (Table 2).

**Table 2:** Example record from the metadata table.

filename	class	candidate_id
Railway_Track_0001.png	railway	Railway_track_0001

For descriptive purposes, each image chip was assigned a contextual environment label derived from OpenStreetMap area geometries [10]. Intersecting areas were grouped into agriculture or open vegetation, urban, forest, industrial, and water or wetland categories based on selected OpenStreetMap tags. The geometries were clipped to the chip footprint and the overlap area was calculated for each category. The category with the largest overlap was assigned to the chip. Chips without qualifying OpenStreetMap area overlap were assigned the label unknown_osm. Table 3 reports the aggregate distribution of these derived labels across the complete image collection and does not constitute reference land-cover data.

**Table 3:** Distribution of contextual environment labels across the image collection.

Environment label	Number of images	Percentage of dataset
agriculture_or_open_vegetation	4,351	43.51
urban	2,846	28.46
forest	2,026	20.26
industrial	554	5.54
water_or_wetland	206	2.06
unknown_osm	17	0.17
**Total**	**10,000**	**100.00**

**Fig. 2:**
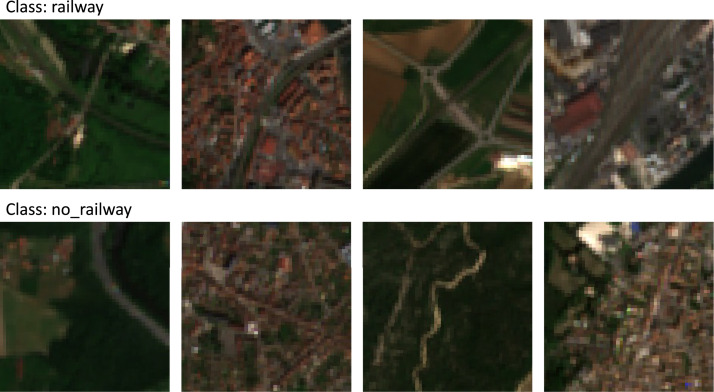
Example Sentinel-2 image chips from the dataset. The panels show selected railway and no_railway image chips across several contextual environment labels. Images are rendered from Sentinel-2 Level-2A B04, B03, and B02 bands [2] and cover 512 m × 512m footprints.

Fig. 2 presents selected image chips from both classes. The figure is intended to illustrate the visual form and spatial extent of the released image data. Each panel shows one 224 × 224 pixel RGB image chip corresponding to a 512 m × 512 m footprint.

To quantitatively describe visual variation in the image collection, four image-level characteristics were calculated for each RGB chip and summarized overall and separately for the railway and railway-absent classes (Table 4). Brightness denotes the mean normalized Value component in the HSV color space, whereas saturation denotes the mean normalized HSV Saturation component. Michelson contrast quantifies the grayscale intensity range using the first and 99th percentiles, reducing sensitivity to extreme pixel values [11]. Canny edge density represents the proportion of pixels identified as edges after grayscale conversion and Gaussian smoothing [12].

**Table 4:** Descriptive image statistics (Average, Avg., and Standard Deviation, SD) for the complete collection and both classes.

Metric	Overall		Railway		No Railway	
	Avg.	SD	Avg.	SD	Avg.	SD
Brightness	0.2111	0.0698	0.2104	0.0638	0.2117	0.0753
Saturation	0.4484	0.0857	0.4416	0.0825	0.4551	0.0882
Michelson Contrast	0.6982	0.1121	0.7094	0.1038	0.6869	0.1187
Canny Edge Density	0.0103	0.0136	0.0108	0.0135	0.0098	0.0137

The results indicate moderately dark and moderately saturated image chips, with comparatively high global grayscale contrast and a low proportion of detected edge pixels. Average values are closely aligned between railway and railway-absent chips across all four measures. This suggests that the two classes are broadly comparable with respect to these global visual characteristics, reducing the risk that classification is driven primarily by class-level differences in brightness, color saturation, contrast, or overall edge content.

## 4 Experimental Design, Materials and Methods

The dataset was collected through a reproducible workflow consisting of four steps: extraction of railway reference geometries from OpenStreetMap [10], spatially balanced selection of railway locations, generation of railway-absent locations, and retrieval of corresponding Sentinel-2 image chips.

Recent publications demonstrate the use of Sentinel-2 imagery for reusable machine-learning datasets, including OpenStreetMap-based road-detection masks, multimodal Sentinel-1 and Sentinel-2 image collections, and cloud and cloud-shadow classification benchmarks [[4], [5], [6]]. These datasets show how transparent acquisition and task-specific labels can support reproducible geospatial modelling. However, no published Sentinel-2 dataset has specifically addressed railway-presence classification through geographically balanced railway sampling and locally generated railway-absent hard negatives. The present workflow addresses this gap by pairing railway scenes with geographically and visually related non-railway examples whose complete footprints are checked for non-intersection with the retained railway network.

Railway geometries and contextual land-cover information were derived from an OpenStreetMap extract covering metropolitan France [10]. The extract was processed locally using pyosmium [13], and only ways tagged railway=rail were retained. Features marked as disused or abandoned, as well as railway service features such as yards, sidings, spurs, and crossovers, were excluded. The retained railway lines were represented both as regularly spaced candidate points and as line segments. The line-segment representation was indexed spatially and later used to test whether a candidate image footprint intersected a retained railway geometry.

Railway samples were selected from the retained railway network with a target of 5,000 locations. Because railway density differs strongly across France, the selection procedure was designed to avoid concentrating samples in dense urban networks or major rail corridors. Candidate points were first distributed across a geographic grid covering the study area and selected in a round-robin sequence across occupied grid cells. A minimum-distance rule was then applied to reduce local clustering between selected locations. Where the initial spacing threshold yielded fewer than 5,000 candidates, the threshold was progressively relaxed according to predefined values until the required number of locations was reached.

Railway-absent samples were generated in the local surroundings of the railway locations. For each railway candidate, the algorithm searched neighboring positions in multiple distance rings and eight compass directions. A railway-absent candidate was accepted only if its complete 512 m × 512 m footprint lay within the study extent, did not overlap a railway footprint, did not overlap an already accepted railway-absent footprint, and had not previously been selected through the spatial grid procedure. Most importantly, the footprint was required not to intersect any retained OpenStreetMap railway segment [10]. This test was performed against the complete indexed line geometry rather than against railway sample points alone. Consequently, a candidate was rejected even when a railway crossed the image footprint without having a resampled railway point located inside that footprint. The final no_railway label therefore denotes the absence of an intersection with the retained OpenStreetMap railway=rail geometries under the stated filtering rules.

For every accepted location, a 512 m × 512 m image chip was retrieved from the Copernicus Sentinel-2 Level-2A collection through the Sentinel Hub Process API of the Copernicus Data Space Ecosystem [14]. Sentinel-2 is a high-resolution optical Earth-observation mission designed for systematic land and coastal monitoring. It operates in a near-polar, sun-synchronous orbit at an altitude of approximately 786 km. The constellation architecture uses satellites phased 180° apart in the same orbit, enabling a nominal revisit interval of approximately five days at the equator and more frequent observations at higher latitudes. During the dataset acquisition period, the available collection included observations from Sentinel-2A, Sentinel-2B, and Sentinel-2C. Depending on acquisition date and data availability, individual image chips may therefore originate from one or more of these platforms [7,8].

Each Sentinel-2 platform carries the MultiSpectral Instrument, MSI, a push-broom imaging sensor that acquires data continuously along the flight direction across a 290 km swath. The MSI records 13 spectral bands spanning wavelengths from 443 nm in the visible spectrum to 2190 nm in the shortwave-infrared spectrum. Four bands are provided at 10 m spatial resolution, including B02 at 490 nm for blue, B03 at 560 nm for green, B04 at 665 nm for red, and B08 at 842 nm for near infrared. Six bands are provided at 20 m resolution, including B05 at 705 nm, B06 at 740 nm, B07 at 783 nm, B8A at 865 nm, B11 at 1610 nm, and B12 at 2190 nm. Three bands are provided at 60 m resolution, including B01 at 443 nm, B09 at 945 nm, and B10 at 1375 nm. The MSI uses 12-bit radiometric quantization and combines visible, near-infrared, red-edge, and shortwave-infrared observations to support land-surface monitoring. Sentinel-2 Level-2A products provide atmospherically corrected bottom-of-atmosphere surface-reflectance observations. For the present dataset, the 10 m bands B04, B03, and B02 were used to render the red, green, and blue channels of each RGB image chip [7,8].

Imagery was requested for the period from 23 June 2025 to 23 June 2026, using a maximum cloud-cover threshold of 10% and the leastCC mosaicking option [3]. This option selects observations with comparatively low cloud contamination from the available acquisitions in the specified time range. Although the output images contain 224 × 224 pixels, the underlying Sentinel-2 bands have a native spatial resolution of 10 m [7]; the output therefore represents a resampled visualization of the 512 m footprint rather than data with a native resolution finer than 10 m. The export workflow used authenticated API requests, bounded parallel processing, retry handling for temporary request failures, PNG validation, and resumable execution. All 10,000 requested image chips were successfully generated.

Each chip was additionally assigned a contextual land-cover label from OpenStreetMap area geometries [10]. OpenStreetMap areas intersecting the chip footprint were grouped into the categories industrial, urban, forest, water or wetland, and agriculture or open vegetation on the basis of selected landuse, natural, building, amenity, aeroway, and man_made tags. For each intersecting area, the polygon geometry was clipped to the rectangular chip footprint before its overlap was calculated. Polygon area was computed with the shoelace formula, a standard geometric procedure that estimates the area enclosed by a polygon from the coordinates of its boundary vertices. Clipping before area calculation ensured that only the part of an OpenStreetMap area located inside the image footprint contributed to the contextual label. Category-specific weights were then applied to the overlap values to prevent smaller but semantically distinctive areas, such as industrial sites or dense urban land use, from being dominated by extensive surrounding land-cover polygons. The category with the largest weighted overlap was assigned as the chip-level environment label. Chips without qualifying OpenStreetMap area overlap were assigned the label unknown_osm.

### 4.1 Baseline image-classification evaluation

To provide an initial reference benchmark for future reuse of the dataset, several established image classification backbones were trained to distinguish railway from railway-absent image chips. This evaluation was included to document the use of the released data in a standard downstream classification task and to provide a common baseline for subsequent comparisons. The evaluated architectures comprised ResNet-50 [15], Swin Transformer V2 Base [16], EfficientNet-B4 [17], and ShuffleNet V2 [18]. The selected models include convolutional and transformer-based architectures with different design principles and model capacities. Each model was adapted for binary classification by replacing its original output layer with a two class classification head.

Model performance was estimated with five-fold stratified cross validation using shuffled folds and a fixed random seed of 42. Stratification preserved the distribution of railway and no_railway labels across the five folds. In each fold, four fifths of the images were used for training and the remaining fifth was used for validation. Training images were augmented by random horizontal flipping, random vertical flipping, and color jittering of brightness, contrast, and saturation. No augmentation was applied to validation images.

All model parameters were optimized jointly for a maximum of 50 epochs using the Adam optimizer [19] with an initial learning rate of 1 × 10⁻⁴, weight decay of 1 × 10⁻⁴, and a batch size of 32. Cross entropy loss was used for binary classification. A cosine annealing learning rate schedule was applied throughout training [20]. Early stopping was triggered when validation accuracy did not improve for eight consecutive epochs. For each fold, the model state with the highest validation accuracy was retained.

The retained checkpoints were evaluated on their respective held out validation folds. The evaluation metrics were computed according to (1), (2), (3), (4), where TP, TN, FP, and FN denote true positives, true negatives, false positives, and false negatives, respectively.

(1) $$Accuracy=\frac{TP+TN}{TP+TN+FP+FN}$$

(2) $$Precision=\frac{TP}{TP+FP}$$

(3) $$Recall=\frac{TP}{TP+FN}$$

(4) $$F1\;score=2\times \frac{Precision\;\times \;Recall}{Precision\;+\;Recall}$$

The reported values in Table 5 are the mean and standard deviation across the five validation folds for the four evaluated backbones.

**Table 5:** Mean validation performance across five folds for each evaluated backbone.

Backbone	Accuracy	Precision	Recall	F1 score
**ResNet-50** [15]	94.57% ± 0.20%	94.97% ± 0.90%	94.14% ± 0.72%	0.9455 ± 0.0018
**Swin V2 Base** [16]	95.97% ± 0.30%	96.08% ± 0.58%	95.86% ± 0.64%	0.9597 ± 0.0030
**EfficientNet-B4** [17]	94.83% ± 0.19%	94.12% ± 0.60%	95.64% ± 0.60%	0.9487 ± 0.0018
**ShuffleNet V2** [18]	91.64% ± 0.58%	91.41% ± 0.23%	91.92% ± 1.40%	0.9166 ± 0.0065

Across the evaluated backbones, Swin V2 Base achieved the highest mean validation accuracy of 95.97% ± 0.30% across the five folds.

**Fig. 3:**
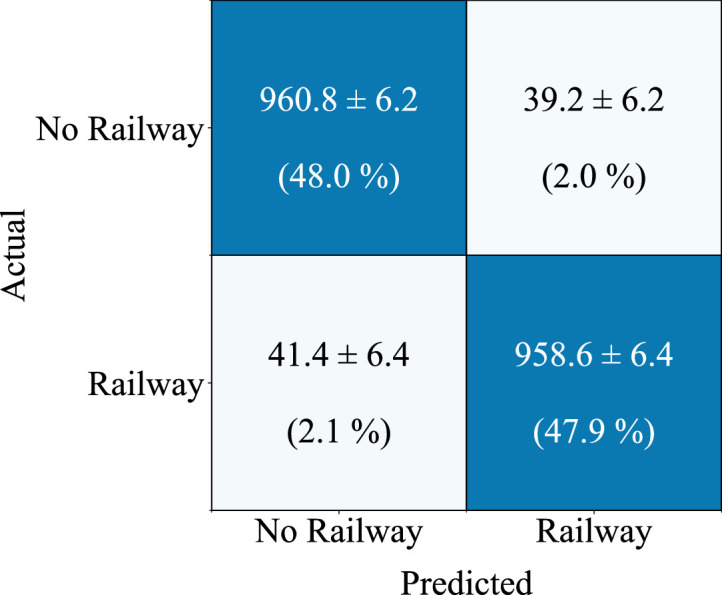
Mean confusion matrix for Swin V2 Base averaged over all five folds.

Fig. 3 presents the confusion matrix for Swin V2 Base aggregated across the held-out validation predictions from all five folds. The matrix reports the number of “No Railway” and “Railway” image chips assigned to each predicted class and provides a class specific view of the classification outcomes.

## Limitations

The railway labels are derived from a specific OpenStreetMap extract [10] and reflect the completeness, positional accuracy, temporal status, and tagging practices of the source data at the time of extraction. In particular, the no_railway label indicates that a chip footprint did not intersect a retained OpenStreetMap railway=rail geometry after the applied filtering procedure. It does not establish the absence of railway related infrastructure that may be missing from, differently tagged in, or inaccurately represented by OpenStreetMap.

The image chips are based on Sentinel-2 B02, B03, and B04 bands with a native spatial resolution of 10 m [7]. The distributed 224 × 224 pixel images are resampled renderings of 512 m × 512 m footprints and do not provide spatial information beyond the native Sentinel-2 resolution. The use of least cloud cover mosaicking reduces cloud contamination but may combine observations from different acquisition dates within the defined temporal window [14].

The dataset covers metropolitan France only and includes railway geometries retained under the specified OpenStreetMap selection criteria [10]. Its geographic coverage and class definitions should therefore be considered when transferring models or comparisons to other countries, time periods, railway categories, or remote sensing products.

## Credit Author Statement

**Lukas Pasold:** Software, Methodology, Validation, Investigation, Data Curation, Visualization, Writing – Original Draft. **Luca Eisentraut:** Methodology, Validation, Formal analysis, Investigation, Writing – Original Draft. **Ricardo Buettner:** Conceptualization, Funding acquisition, Methodology, Project administration, Supervision, Writing – Review & Editing.

## Ethics Statement

The authors have read and follow the ethical requirements for publication in Data in Brief. The present work does not involve human participants, animal experiments, or data collected from social media platforms. The dataset was generated from publicly available OpenStreetMap data [10] and Copernicus Sentinel-2 satellite imagery [7].

## Data Availability

(Mendeley Data).A Sentinel-2 Dataset for Railway Presence Classification Across Metropolitan France (Original data)
